# Management and Outcomes of Isolated Distal Deep Vein Thromboses: A Questionable Trend toward Long-Lasting Anticoagulation Treatment. Results from the START-Register

**DOI:** 10.1055/s-0041-1730038

**Published:** 2021-07-08

**Authors:** Gualtiero Palareti, Cristina Legnani, Emilia Antonucci, Sophie Testa, Daniela Mastroiacovo, Benilde Cosmi, Daniela Poli, Eugenio Bucherini, Francesco Dentali, Andrea Fontanella, Nicola Mumoli, Davide Imberti, Anna Falanga, Walter Ageno, Fulvio Pomero

**Affiliations:** 1 Arianna Anticoagulazione Foundation, Bologna, Italy; 2 Centro Emostasi e Trombosi A O Istituti Ospitalieri di Cremona, Cremona, Italy; 3 Daniela Mastroiacovo MD, Dipartimento di Angiologia, Ospedale SS. Filippo e Nicola, Avezzano, (L'Aquila), Italy; 4 Department of Angiology and Blood Coagulation, University Hospital, Bologna, Italy; 5 SOD Malattie Aterotrombotiche, Azienda Ospedaliero Universitaria-Careggi, Firenze, Italy; 6 SS Aziendale di Angiologia Faenza AUSL Romagna, Ravenna, Italy; 7 Dipartimento di Medicina Interna, Ospedale di Circolo, Università dell'Insubria, Varese, Italy; 8 Dir. Dipartimento di Medicina, Ospedale del Buon Consiglio-Fatebenefratelli, Napoli, Italy; 9 Department of Internal Medicine, Magenta Hospital, Magenta (MI), Italy; 10 UO Medicina Interna ERI, Ospedale Civile, Piacenza, Italy; 11 Università Milano Bicocca, Dipartimento di Medicina e Chirurgia, Italy; 12 UOC Immunoematologia e Medicina Trasfusionale ASST Bergamo, Italy; 13 Dipartimento di Emergenza e Accettazione, Centro Trombosi ed Emostasi, Ospedale di Circolo, Università dell'Insubria, Varese, Italy

**Keywords:** isolated distal deep vein thrombosis, oral anticoagulant, bleeding, duration of treatment

## Abstract

**Background** Isolated distal deep vein thromboses (IDDVT) are frequently diagnosed; however, their natural history and real risk of complications are still uncertain. Though treatment is still not well standardized, international guidelines recommend no more than 3 months of anticoagulation therapy. We investigated how Italian clinicians treat IDDVT patients in their real life in our country.

**Methods** Baseline characteristics and clinical history of the patients enrolled in the prospective, observational, multicenter START-Register for a first IDDVT or proximal DVT (PDVT) were analyzed.

**Results** Overall, 412 IDDVT patients were significantly younger, with better renal function, and more frequent major transient risk factors, when compared with 1,173 PDVT patients. The anticoagulation duration was >180 days in 52.7% of IDDVT patients (70.7% in PDVT). During treatment, bleeding occurred in 5.6 and 2.8% patient-years in IDDVT and PDVT, respectively ( *p* = 0082). Bleeding was more frequent in IDDVT than PDVT patients treated with warfarin (6.8 vs. 3.2 patient-years, *p* = 0.0228, respectively). Thrombotic complications occurred in 1.1 and 2.4% patient-years in IDDVT and PDVT patients, respectively. Analyzing together the two groups, 66.1% of bleeds and 86.1% thrombotic complications occurred after 90 days anticoagulation treatment.

**Conclusion** The large majority of IDDVT patients received anticoagulation for more than 3 months. Most bleeding and thrombotic complications occurred after the first 90 days of anticoagulation therapy. These results indicate that an extended anticoagulation beyond 90 days in IDDVT patients is associated with increased risk of complications. Whether an extended treatment may lower recurrences after anticoagulation withdrawal should be assessed by specifically designed studies.

## Introduction

Isolated distal deep vein thrombosis (IDDVT) generally refers to a thrombotic process that affects one or more of the deep calf veins (either axial or muscular) but does not involve the popliteal or more proximal veins (PDVT). The presence of IDDVT is a frequent finding in patients with suspected DVT; however, its prevalence in suspected patients varies greatly, 1 mainly due to the possible adoption of different ultrasound diagnostic procedures which may be limited to just proximal deep veins or else extend to calf veins (thus potentially able to identify distal DVTs). 2 Once IDDVTs are diagnosed, their optimal treatment is far from being standardized and long-term clinical outcomes unclear. 3 For this disease, heparin or derivatives (especially low molecular weight heparin [LMWH]), vitamin-K antagonists (VKAs), and direct oral anticoagulants (DOACs) can be used for therapy. Currently, however, optimal treatment has still not been adequately standardized: anticoagulant of choice, appropriate dosing, and duration of treatment are still a matter of discussion. This state of affairs, in large part, can be put down to a general lack of interest by researchers in the disease, as witness the literature which has been persistently much less than that on proximal DVT or pulmonary embolism. 4 Given the relative paucity of randomized clinical trials, prospective observational studies can give us important information on clinicians' approach to diagnosis, management, and treatment of IDDVT.

The present study aimed at analyzing the therapeutic approach of Italian clinicians to patients diagnosed with acute IDDVT, as first venous thrombotic episode, who were included in the prospective, observational, multicenter START-VTE-Registry. Their baseline characteristics, type of management, and clinical results were analyzed and compared with those recorded in patients included in the registry for a first event that was proximal DVT of a lower limb.

## Materials and Methods

### The START-Register

The START-Register (ClinicalTrials.gov identifier: NCT02219984) is an observational, multicenter, dynamic cohort study of adults (≥18 years) starting anticoagulation therapy, whatever the indication for treatment and drug/dosage used. 5 The present article reports only on patients included in the registry for a first venous thrombotic event due to IDDVT or proximal DVT (PDVT). Authorization to set up the START-Register was obtained from the Ethical Committee of the University Hospital “S. Orsola-Malpighi,” Bologna, Italy, on October 2011 (no.: 142/2010/0/0ss”) which is entrusted with the deployment and upkeep of the registry central database. The registry is one of the activities of the “Arianna Anticoagulazione” Foundation (Bologna, Italy). The registry is open to all physicians (called participants) prescribing anticoagulant or antithrombotic therapy who agree to the registry protocol. Participants should obtain approval from their local institution review board and are required to enroll their patients consecutively or randomly, with no exclusion criteria other than obstacles for follow-up (short life-expectancy or geographical inaccessibility). Participants enroll patients only after receiving their informed consent. The participating centers are required to enroll their patients consecutively, without any a priori exclusion criteria other than life-expectancy or geographical inaccessibility. Definition of the time frame for enrolment (e.g., 1 week every month or the first month of the year) is left at each participant's discretion, as long as it provides a random enrolment of patients. The accuracy and completeness of the data collected anonymously in the central electronic database are checked by a trained and dedicated monitor figure who also solicits participating centers to contact, for the purpose of this study, patients lost to follow-up either by a telephone call or through their General Practitioner. The study was performed and is reported according to the Strengthening the Reporting of Observational Studies in Epidemiology (STROBE) guidelines for observational studies. 6

### Study Population

All patients with clinical indications of DVT and/or pulmonary embolism (PE) were registered in the START-Register, including the FADOI-START-Register (which is a subportion of the START-Register dedicated to VTE patients included by centers affiliated with the Italian FADOI Federation [“Federazione delle Associazioni dei Dirigenti Ospedalieri Internisti”]).

The present study focused on patients included in the START-Register for October 2010 up to June 2018 for a first episode of IDDVT or of PDVT of a lower limb; their baseline characteristics, anticoagulation management, and clinical results were compared. We limited the study to patients whose VTE event was the first in their life since our focus was on the type of treatment decision taken in patients with no VTE history. Patients included in the registry for a PE event (either in isolation or associated with PDVT or IDDVT) were also excluded from the study, since this condition is generally considered a more serious disease that is likely to have an influence on the therapeutic approach of treating physicians. The treatments and the events occurring during follow-up were then analyzed in IDDVT and PDVT patients. LMWHs, fondaparinux, and warfarin (the VKA used in approximately 99% of considered patients) were always available as anticoagulant drugs for therapy during the observation period, whereas DOACs have only been allowed and reimbursed by the National and Regional Health Systems for initial, long term and extended anticoagulant treatment for patients with venous thromboembolism (VTE; including IDDVT) since January 2014.

### Data Collection

Participants in the START-Register connect to the central electronic database via web using individual passwords. Information is recorded in structured case report form (CRF) and involves baseline characteristics of included patients and their follow-up. Baseline characteristics of patients included in the present study were (1) demographic, body weight, routine laboratory data, and past medical history (hypertension, diabetes mellitus, heart failure, coronary arteries disease, peripheral arteries disease, atrial fibrillation, previous stroke or transient ischemic attack or systemic embolism, chronic pulmonary disease, gastrointestinal disease, thyroid disease, previous clinically relevant bleeding, malignancy, renal and liver function, and alcohol abuse); (2) characteristics of index VTE event and presence of risk factors; (3) anticoagulant agents used, dosages, or the intended therapeutic range (2.0–3.0 international normalized ratio [INR] in all cases receiving VKAs), concomitant medications (especially antiplatelet drugs).

Serum creatinine levels were measured by local hospital laboratories and creatinine clearance (CrCl) calculated by the Cockcroft and Gault equation. 7 Renal failure was defined according to National Kidney Foundation stratification. 8

Information was collected about the nature and site of VTE events. The nature of index event was considered as follows: (1) unprovoked, when not temporally associated with any potential triggering conditions or risk factors (RF); (2) associated with weak RF, such as minor, arthroscopic or laparoscopic general surgery, pregnancy or puerperium, contraceptive or replacement hormonal therapy, long trip, minor trauma, stay in hospital, and reduced mobility (not complete immobilization); (3) provoked by transient major RF when in association with one of the following conditions occurring within 3 months of VTE diagnosis: major surgery with general or spinal anesthesia, lower limb fracture, casting or no weight bearing for ≥3 days, bed-bound for >3 days due to acute illness, and others; (4) provoked by permanent major RF, when associated with active cancer, paraplegia, chronic active inflammatory disease (e.g., intestinal inflammatory disease) or other chronic serious diseases, serious inherited thrombophilic alterations, antiphospholipid syndrome, severe postthrombotic syndrome, and presence of cava filter.

For the present analysis, the site of events was classified in (1) cases with IDDVT, when thrombosis involved only calf veins and was not associated with diagnosis of PE; or (2) PDVT, when thrombosis involved popliteal or onward deep veins (with or without calf DVT), without PE diagnosis. The patients included in the registry with PE, with or without PDVT or IDDVT, were not included in the present analysis.

### Follow-up Data

The present study reports on the events occurred in IDDVT and PDVT patients during their anticoagulant treatment. Follow-up was considered from inclusion of patients until December 2018, or until a permanent cessation of anticoagulant treatment, the last follow-up available in patients who subsequently were lost to follow-up or declined to further participate in the START-Register, or occurrence of major bleeding, thrombotic complications, death, whichever came first. During follow-up, detailed clinical reports of any relevant clinical outcome occurring in enrolled patients were collected. Major bleeding (MB), was defined according to International Society on Thrombosis and Haemostasis criteria. 9 Clinically relevant nonmajor bleeding (CRNMB) events were defined as any overt bleeding requiring a medical intervention and/or treatment discontinuation, not meeting any of the criteria for major bleeding. 10 Thromboembolic events were recorded and defined as verified events of either venous type (recurrent VTE episode, venous thrombosis in different sites, or superficial vein thrombosis) or arterial type (stroke/arterial thromboembolism/TIA or myocardial infarction).

### Statistical Analysis

Continuous variables are expressed as median with interquartile range (IQR). Categorical variables are expressed as frequencies and percentages. The number of bleeding events was expressed as percentage (with 95% confidence intervals [CI]) and incidence rate and calculated as the number of events per 100 patient-years of observation. Differences between groups were assessed using the ×2 test with Yates' correction for categorical variables and the Mann–Whitney *U* -test for continuous variables. The data were analyzed with the use of Prism software (Version 8.4.0, GraphPad Software Incorporated, San Diego, California, United States), and Stata, version 14 statistical software package (Stata Corp.) was used for data processing.

## Results

**Fig. 1:**
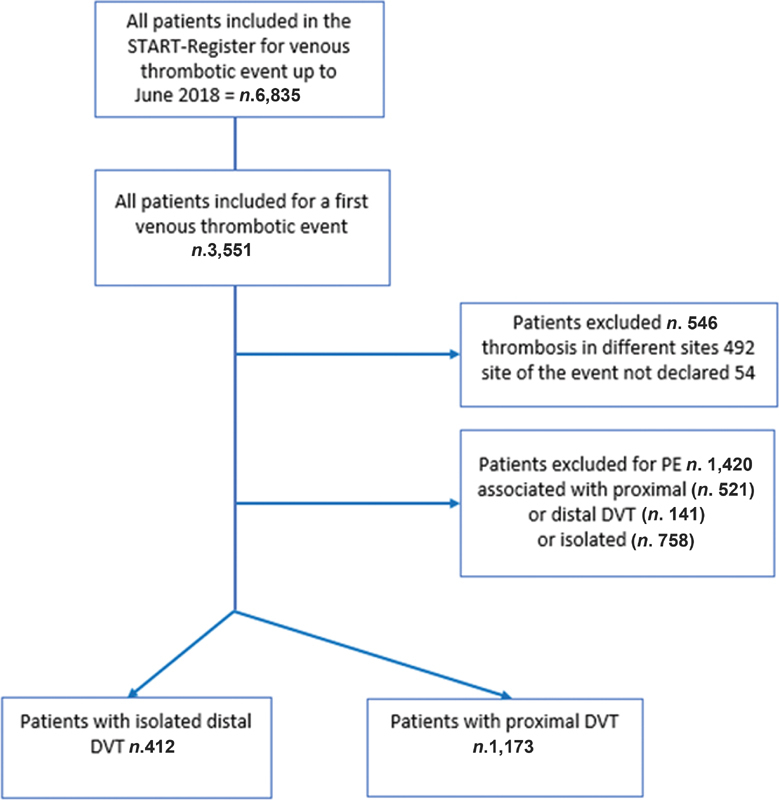
Patient flowchart. DVT, deep vein thrombosis; isolated distal DVT: thrombosis of one or more calf deep veins not involving the popliteal vein; PDVT: DVT that involves the popliteal vein and/or more proximal deep veins (with or without distal DVT); PE: pulmonary embolism.

As shown in Fig. 1 , from the 6,835 patients included in the START-Register up to June 2018 for occurrence of one or more venous thromboembolic events, 3,551 patients presenting first-in-life event were identified. After excluding 1,966 patients (492 for thrombosis in different sites, 54 because the site of the event was not reported, and 1,420 because the event was a PE), 412 patients with IDDVT were analyzed and compared with the 1,173 with PDVT who were enrolled in the START-Register by 51 clinical centers which on average included 26 patients (minimum = 3, maximum = 301).

## Baseline Characteristics of Patients

At baseline ( Table 1 ), patients with IDDVT versus PDVT were significantly younger with a lower presence of elderly (≥75 years), had a generally better renal function, and a lower prevalence of so-called fragile conditions. The unprovoked or provoked nature of thrombotic events was equally distributed across the two groups of patients; however, major risk factors were more frequently transient in IDDVT ( *p* = 0.0001) and permanent in PDVT ( *p* < 0.0001). Less patients with IDDVT were fragile ( *p* = 0.0002) or had chronic inflammatory diseases ( *p* = 0.0237), whereas more of them were treated for hypertension ( *p* = 0.0193). Users of lipid lowering drugs were more frequent among IDDVT ( *p* = 0.0045).

**Table 1:** Baseline characteristics of patients with a first event of IDDVT or PDVT

*n*	IDDVT ( *n* = 412)	PDVT ( *n* = 1,173)	*p*
Male, *n* (%)	205 (49.8)	619 (52.8)	0.2945
Age (y) Median (IQR)	65 (49.2–75)	69 (53–79)	0.0005
Age classes (y) *n* (%)			
<60	162 (39.3)	384 (32.7)	0.0153
60–74	140 (34.0)	356 (30.3)	0.1635
≥75	110 (26.7)	433 (37.0)	0.0002
BMI (kg/m ^2^ ) Missing	26 (24–29) 3	26 (24–29) 9	0.7403
First available creatinine Median (IQR)	0.90 (0.70–1.00)	0.90 (0.80–1.00)	0.0161
Creatinine >1.5 mg/dL *n* (%)	16 (3.9)	55 (4.7)	0.5002
CrCl *n* (%)			
< 30 mL/min	9 (2.2)	29 (2.5)	0.7334
30–59 mL/min	82 (19.9)	321 (27.4)	0.0027
≥60 mL/min	321 (77.9)	821 (70.1)	0.0024
Missing	-	3	
Nature of VTE events *n* (%)			
Unprovoked	260 (63.3)	746 (63.6)	0.9134
Provoked	151 (36.7)	427 (36.4)	
• By weak RFs	24 (16.0)	53 (12.4)	0.2639
• By transient major RFs	92 (60.9)	180 (42.2)	0.0001
• By permanent major RFs	35 (23.1)	194 (45.4)	<0.0001
• Cancer	19 (12.6)	80 (18.7)	0.0874
• Missing	1	-	
Diabetes	35 (8.5)	118 (10.1)	0.3450
Hypertension	115 (27.9)	260 (22.2)	0.0193
IHD, CVD, PAD	60 (14.6)	174 (14.8)	0.9216
Heart failure	11 (2.7)	35 (3.9)	0.2601
Chronic inflammatory dis	44 (10.7)	178 (15.2)	0.0237
Known thrombophilia	33 (8.0)	116 (9.9)	0.2559
Fragile (age > 75 y, or ≤50 kg, or CrCl <50 mL/min)	122 (29.6)	470 (40.1)	0.0002
Associated treatments			
• Antiplatelet drugs	35 (8.5)	89 (7.6)	0.5588
• Antiarrhythmic drugs	7 (1.7)	23 (2.0)	0.7029
• Antidiabetics	27 (6.6)	79 (6.7)	0.9442
• Lipid lowering drugs	66 (16.0)	126 (10.7)	0.0045
Anticoagulation treatments			
• LMWHs *n* (%)	26 (6.3)	73 (6.2)	0.9424
Fondaparinux *n*	9	31	
Duration (mo) Median (IQR)	2.8 (0.9–6.5) a	6.9 (3.1–12.8)	
• Warfarin *n* (%)	185 (44.9)	381 (32.5)	<0.0001
Duration (mo) Median (IQR)	9.6 (4.3–23.1)	14.5 (6.8–26.3)	
• DOACs *n* (%)	201 (48.8)	719 (61.3)	<0.0001
Duration (mo) Median (IQR)	5.6 (3.1–9.7) b	8.7 (4.7–15.9)	
Apixaban *n* ( *n* . low dose)	38 (6)	157 (33)	
Dabigatran *n* ( *n* . low dose)	28 (3)	72 (10)	
Edoxaban *n* ( *n* . low dose)	15 (1)	55 (17)	
Rivaroxaban *n* ( *n* . low dose)	120 (0)	435 (24)	

Abbreviations: BMI, body mass index; CrCl, creatinine clearance; CVD, cerebrovascular disease; DOACs, direct oral anticoagulants; IDDVT, isolated distal deep vein thrombosis; IHD, ischemic heart disease; IQR, interquartile range; LMWH, low molecular weight heparin; PAD, peripheral artery disease; PDVT, proximal deep vein thrombosis; RFs, risk factors; VTE, venous thromboembolism.

a The duration of treatment with parenteral drugs was significantly shorter than that with warfarin or DOACs ( *p* < 0.0001).

b The duration of treatment with DOACs was significantly shorter than that with Warfarin ( *p* = 0.0103).

Parenteral anticoagulation drugs (including LMWH or fondaparinux) were used in a small and similar proportion in IDDVT and PDVT patients (∼6%). Warfarin and DOACs were prescribed in equal measure in IDDVT patients, whereas DOAC use was markedly higher and warfarin lower in PDVT patients (61.3 and 32.5%, respectively). Among DOACs, rivaroxaban was the most frequently used drug, given it was the first in the category to be available for this indication (since 2014) and reimbursed by the Italian National Healthcare System. In both IDDVT and PDVT groups, the treatment was shortest in patients using parenteral anticoagulants and longest in those receiving warfarin ( Table 1 ).

### Duration of Anticoagulation and Outcomes during Treatment

As reported in Table 2 , less than one-fourth (22.3%) of IDDVT patients received anticoagulation treatment up to 90 days, and more than half (52.7%) were treated for >180 days. About 29% of PDVT patients were treated for up to 180 days, whereas the large majority (>70%) received anticoagulation for >180 days. Among IDDVT patients, males were treated longer than women, with only 38% of them being treated for up to 90 days versus 62% of women. Patients with an unprovoked event received anticoagulation for >180 days (70.5%). More patients, anticoagulated with warfarin, were treated for >180 days, as well as the patients with known thrombophilic alterations. Since it was plausible to expect that the use of DOACs for VTE indication, starting in our country on 2014, could have affected the results of treatment of patients, we performed a subanalysis of treatment characteristics in IDDVT patients by comparing those included between 2010 and 2014 ( *n* = 208) or between 2015 and 2018 ( *n* = 204). The VKAs use dropped from 77.4 to 11.3% ( *p* < 0.0001), whereas DOACs increased from 20.7 to 78% ( *p* < 0.0001); at the same time, the median duration of treatment lowered from 8.7 months (IQR: 4.4–23.4) to 4.9 months (IQR: 3.0–9.1; *p* < 0.0001). Similar results were found also in patients with PDVT.

**Table 2:** Distribution of treatment duration in patients with IDDVT or PDVT in relation to the anticoagulant drug used and characteristics of IDDVT patient

Duration of anticoagulation *n* (%)	≤90 days	91–180 days	>180 days	*p*
IDDVT patients	92 (22.3)	103 (25.0)	217 (52.7)	<0.0001
LMWH-treated ( *n* = 26)	13	6	7	
VKA-treated ( *n* = 185)	33	36	116	
DOAC-treated ( *n* = 201)	46	61	94	
PDVT patients	144 (12.3)	200 (17.0)	829 (70.7)	<0.0001
LMWH-treated ( *n* = 73)	18	13	42	
VKA-treated, ( *n* = 381)	34	43	304	
DOAC-treated ( *n* = 719)	92	144	483	
Characteristics of IDDVT patients				
Males	35 (38.0)	45 (43.7)	125 (57.6)	0.0026
Females	57 (62.0)	58 (56.3)	92 (42.4)	
Nature of index events				
Unprovoked	50 (54.3)	58 (56.3)	153 (70.5)	0.0258
Provoked (weak and transient RFs)	34 (37.0)	33 (32.0)	49 (22.6)	
Cancer or other permanent RFs	8 (8.7)	12 (11.7)	15 (6.9)	0.3636
Age (y)				
< 60	37 (40.2)	41 (39.8)	84 (38.7)	0.9631
≥60	55 (59.8)	62 (60.2)	133 (61.3)	
First available CrCl (mL/min)				
< 60	23 (25.0)	22 (21.4)	46 (21.2)	0.7464
≥60	69 (75.0)	81 (78.6)	171 (78.8)	
BMI (kg/m ^2^ )				
≤25	35 (38.0)	38 (36.9)	69 (31.8)	0.4742
26–30	45 (49.0)	49 (47.6)	103 (47.5)	
> 30	12 (13.0)	16 (15.5)	25 (20.7)	
Fragile (age > 75 y, or ≥50 kg, or CrCl <50 mL/min)	35 (38.0)	26 (25.2)	61 (28.1)	0.1156
Diabetes	8 (8.7)	5 (4.9)	22 (10.1)	0.2844
IHD, CVD, PAD	7 (7.6)	19 (18.4)	34 (15.7)	0.0806
Heart failure	3 (3.3)	3 (2.9)	5 (2.3)	0.8787
Chronic inflammatory diseases	11 (12.0)	7 (6.8)	26 (12.0)	0.3377
Known thrombophilia	2 (2.2)	4 (3.9)	27 (12.4)	0.0020
Anticoagulant drug used				
LMWH/Fondaparinux	13 (14.1)	6 (5.8)	7 (3.2)	0.0001
Warfarin	33 (35.9)	36 (35.0)	116 (53.5)	
DOACs	46 (50.0)	61 (59.2)	94 (43.3)	

Abbreviations: BMI, body mass index; CrCl, creatinine clearance; CVD, cerebrovascular disease; DOACs, direct oral anticoagulants; IDDVT, isolated distal deep vein thrombosis; IHD, ischemic heart disease; LMWH, low molecular weight heparin; PAD, peripheral artery disease; PDVT, proximal deep vein thrombosis; RFs, risk factors.

Note: Statistically significant *p* -values (< 0.05) are depicted in bold

During treatment ( Table 3 ), 21 (5.6% patient/years) bleeding events (either major or clinically relevant nonmajor) occurred in IDDVT and 38 (2.8% patient/years) in PDVT patients ( *p* = 0.0082). In both groups, the incidence of bleeding events was higher (though not statistically significant) in patients receiving warfarin (4.2% patient/years) than DOACs (2.6% patient/years, *p* = 0.0717); it was significantly higher in IDDVT than PDVT patients who received warfarin ( *p* = 0.0228). Four (1.1% patient-years) thrombotic events occurred during treatment in IDDVT and 32 (2.4% patient-years) in PDVT patients (not significant). The incidence of thrombotic complications was similar in IDDVT or PDVT warfarin-treated patients (1.3 vs. 0.9% patient/years, respectively), and lower, though still not statistically significant, in DOAC-treated IDDVT versus PDVT patients (0.7 vs. 3.2% patient/years, respectively). Bleeding and thrombotic complications occurred more frequently late during treatment in both groups of patients. Taking the two groups together, 66.1% of bleeds and 86.1% of thrombotic complications occurred after 90 days of anticoagulant treatment

**Table 3:** Bleeding and thrombotic complications occurred during treatment in patients with IDDVT or PDVT

	IDDVT n. 412		PDVT n. 1,173		
Total follow-up (y)	376		1352		
Median FU (IQR) in months	6.2 (3.2–15.6)		10.0 (5.4–19.5) a		
Major and CRNMB *n* (% patient-years)	21 (5.6)		38 (2.8) b		
LMWHs/fondaparinux	0		3 (5.1)		
Warfarin	15 (6.8)		19 (3.2) c		
DOACs	6 (4.3)		16 (2.2)		
Timing of bleeding events *n* (% patient-years)					
≤90 days (IDDVT = 11 y; PDVT = 17 y)	6 (54.5)		14 (82.3)		
91–180 (IDDVT = 35 y; PDVT = 68 y)	6 (17.1)		5 (7.3)		
>180 (IDDVT = 220 y; PDVT = 1,209 y)	9 (4.1)		19 (1.6)		
Site of bleeds ( *n* )	Warfarin	DOACs	LMWHs	Warfarin	DOACs
ICH	-	1	-	1	-
GIH	1	2	2	1	2
Other	14	3	1	17	14
Thrombotic events *n* (% patient-years)	4 (1.1)		32 (2.4)		
LMWH/fondaparinux	0		4 (6.9)		
Warfarin	3 (1.3)		5 (0.9)		
DOACs	1 (0.7)		23 (3.2)		
Timing of thrombotic events *n* (% patient-years)					
≤90 days (IDDVT = 11 y; PDVT = 17 y)	-		5 (29.4)		
91–180 (IDDVT = 35 y; PDVT = 68 y)	-		3 (4.4)		
>180 (IDDVT = 220 y; PDVT = 1,209 y)	4 (1.8)		24 (2.0)		
Type of thrombotic events	Warfarin	DOACs	LMWHs	Warfarin	DOACs
Venous	1	-	4	5	20
Arterial	2	1	-	-	3
Deaths during follow-up *n* (%)	13 (3.1)		82 (7.0)		
Cancer, IHD, other (n)	6, 3, 4		28, 10, 44		
Lost to follow-up *n* (%)	4 (1.0)		9 (0.8)		

Abbreviations: CRNMB, clinically relevant non-major bleeding; DOACs, direct oral anticoagulants; FU, follow-up; GIH, gastrointestinal hemorrhage; ICH, intracranial hemorrhage; IDDVT, isolated distal deep vein thrombosis; IHD, ischemic heart disease; IQR, interquartile range; LMWH, low molecular weight heparin; PDVT, proximal deep vein thrombosis; y, years.

a The median duration of follow-up was significantly longer for PDVT than IDDVT patients ( *p <* 0.0001).

b The bleeding incidence was higher in IDDVT than in PDVT patients ( *p* = 0.0082).

c The bleeding incidence was higher in IDDVT than PDVT warfarin treated patients ( *p* = 0.0228).

**Fig. 2:**
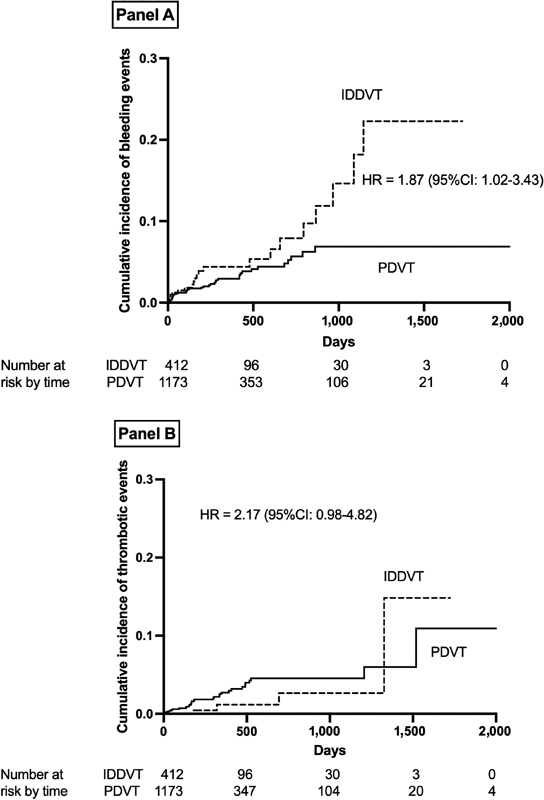
Cumulative occurrence of bleeding ( **Panel A** ) and thrombotic ( **Panel B** ) events in patients with isolated distal (IDDVT) or proximal (PDVT) deep vein thrombosis. CI, confidence interval; HR, hazard ratio.

The cumulative occurrence of bleeding (Panel A) and thrombotic (Panel B) complications during treatment are shown in Fig. 2 . The hazard ratio (HR) of bleeding events in IDDVT versus PDVT was 1.87 (95% CI: 1.02–3.43); the incidence of thrombotic events was not significantly different between the two groups.

## Discussion

Important results of the study are (1) the large majority of IDDVT patients received anticoagulation for more than the 3 months suggested by international guidelines; furthermore, more than half of them were anticoagulated for >180 days, a therapeutic decision taken more frequently in males when the event was unprovoked, patients with known thrombophilic conditions, and patients treated with warfarin; (2) such a long period of anticoagulation was associated with a nonnegligible occurrence of complications, especially bleeding (more than 70% of all bleeds occurred after the first 90 days of treatment); (3) the use of DOACs instead of VKAs for anticoagulation therapy in IDDVT patients increased sharply from 20.7% (during the period 2010–2014) to 78% (during 2015–2018) and was associated with a significant reduction of median duration of treatment (from 8.7–4.9 months).

We were surprised to see how long Italian physicians treat patients with a first IDDVT episode with anticoagulants. Only a minority of these patients (22%) were treated for a maximum of 3 months, whereas more than half received extended treatment. Albeit with some differences, recent international studies seem to support the view that giving long anticoagulant treatment to IDDVT patients is a growing trend, not limited to Italian physicians. Ageno & Co. 11 found that only 27.1% of IDDVT patients included in the prospective XALIA study had a treatment duration of up to 90 days, whereas 27.9% of them were treated for >180 days. Schellong & Co. 12 analyzed IDDVT patients who were included in the prospective GARFIELD-VTE study and found that 60% of them were anticoagulated at 6 months of follow-up and 40% at 12 months of follow-up.

Certainly, IDDVT is a condition that has long been object of debate. 3 Since the natural history of IDDVT is still uncertain and the risks associated with it not clearly quantified, even the need to test for distal DVT in patients with suspected DVT after exclusion of PDVT remains a matter of debate, with different approaches taken in clinical practice. 13 It was clearly shown that IDDVT is associated with a lower risk of recurrence than PDVT or PE, 14 15 16 17 while most untreated IDDVT have a benign clinical course with only some of them having clinically relevant thrombotic outcomes. 18 Moreover, the two studies 19 20 that randomized symptomatic patients to two different diagnostic procedures, allowing and not allowing diagnosis (and treatment) of IDDVT, concluded that the two strategies provided similar results in terms of thrombotic complications after 3 months of follow-up, thus proving it was not necessary to diagnose (and treat) IDDVT. Although these and other data clearly question the need to test for distal DVT, 21 after IDDVTs are diagnosed the issue of their optimal treatment emerges, and especially how long anticoagulation therapy should last. In fact, a shorter duration of anticoagulation in these patients is a crucial point to lower the risk of bleeding, necessarily associated with anticoagulation, found in the present and other studies. 22 Too short anticoagulation treatments, such as 4 to 6 weeks, offered inadequate protection against recurrence. 23 International guidelines 13 and experts 24 have recommended 3 months of anticoagulation for IDDVT patients (the same duration recommended for patients with proximal DVT and/or PE) without extension of treatment.

Despite these recommendations, the present and other real-life studies (mentioned above) clearly show that there is a general trend lengthen anticoagulation treatment periods, not only for unprovoked major venous thrombotic events (such as proximal DVT and/or PE) but also for less serious events such as IDDVT. Today clinicians seem less inclined to set optimal duration periods for anticoagulation of VTE patients based on the balance between thrombotic risk and potential side effects of a long-lasting treatment. The reasons for this trend are many. First of all, deciding on how long anticoagulation should last in each patient is still a complex and far from easy issue to resolve for physicians; furthermore, the time and resources physicians and clinical centers have at their disposal for this task have been steadily declining in recent years, at least in our country. Many physicians and clinical centers prefer (or are forced by conditions) to continue treatment instead of calling in patients for a visit to decide whether it is preferable to stop anticoagulation or not. Some clinicians fear recurrence if anticoagulants are withdrawn and feel confident that currently available anticoagulants are safe enough to continue treatment indefinitely. An example of the effects of resource limitation can be seen in our results where IDDVT patients treated with warfarin had the longest period of treatment. The majority of these patients were managed by START-participants who work in anticoagulation clinics. In current working conditions, it is much easier for managing physicians to stick to their usual routine work (INR control and next dose prescription) than to try and fix an appointment for a possible stop therapy, inform the patient about the need for this step, find a space and a doctor available for a visit, etc. In this way, treatments are continued, and possible discontinuation of therapy simply not taken into consideration which clearly is a problem of organization and resources. The situation is different with DOAC use in Italy, since the National Health Service still requires a prescription in every 12 months (or 6 months for VTE in some regions) if the drugs are to be reimbursable. It is likely, therefore that VTE patients will receive DOAC treatment for periods of 6 or 12 months, or multiples. Our study shows that after their introduction for VTE treatment in 2014, the use of DOACs has rapidly increased in our country up to almost completely replace VKAs. Interestingly, DOACs use was associated with a marked reduction in the duration of anticoagulation treatment.

Other interesting results in our study are mentioned hereinafter. In keeping with previous reports, 25 26 27 28 we found a trend for more bleeding complications in both distal and proximal DVT patients treated with warfarin than with DOACs. Among the patients treated with warfarin, bleeding events were significantly more frequent in IDDVT than in PDVT patients. It is well known that the bleeding risk with warfarin is higher during the first months of treatment, 29 our finding can therefore be attributed to the shorter therapy in IDDVT than PDVT patients. Whatever the drug used, our study showed that many bleeding complications occurred late in treatment (>90 days), a finding that would seem to discourage extending anticoagulation in subjects who are not at high risk of recurrence. Furthermore, all the few thrombotic events involving IDDVT patients and the majority of those in proximal DVT patients occurred late during treatment (>180 days). In our opinion, this finding, which is in line with what we have recently published in patients with venous thromboembolism, 30 has little to do with the efficacy of the drugs, but mainly to patients not adhering as well to treatment. The result highlights the importance of regular periodic visits during extended therapy in DOAC-treated patients to hammer home the need to stick to therapy and to lower the occurrence of late treatment failure.

## Limitations

The study has limitations. There is a large difference among the number and observation period of the two investigated groups of patients. All the data analyzed were collected in a prospective observational registry in which all the therapeutic decisions were left to the attending physicians. During the time interval of the study, there was a progressive increase of DOACs prescription, with a changing proportion of VKA-treated patients over time, a fact that may have been a confounder. Finally, during the study, it was impossible to collect data on the adherence of DOAC-treated patients to the prescribed therapeutic regimens; the persistence to treatment, however, was confirmed.

## Conclusion

In conclusion, we found that the large majority of IDDVT patients received anticoagulation for more than the first 3 months after the event (>50% of patients received anticoagulant treatment for >180 days). This long treatment, which is in contrast with current recommendations in international guidelines, was associated with bleeding events (>70% of all bleeds occurred after the first 90 days of treatment) and also thrombotic complications during anticoagulation. These results lead us to comment that the standard 3 months anticoagulation period seem to be indicated for the majority of IDDVT patients. Specifically designed prospective studies are needed to assess the balance between the risks associated with an extended treatment beyond 90 days in these patients and the potential advantages on the rate of recurrences after anticoagulation is stopped.

## Notes

**What Is Known on This Topic**

- The presence of an isolated distal deep vein thrombosis (IDDVT) is frequent in suspected DVT patients; however, its natural history, the associated clinical risks, and the optimal treatment are still uncertain.
- International guidelines recommend not more than 3 months of anticoagulation in patients with IDDVT.
- Which is the physicians' practice in real-life conditions in our country is not known.

**What Does This Paper Add**

- From the observational, prospective, multicenter START-Register, we examined 412 patients included for a first isolated distal deep vein thrombosis (IDDVT) event and compared with 1,173 patients with a first proximal DVT.
- The large majority of IDDVT patients received anticoagulation for more than the recommended 3 months, and more than half for more than 6 months.
- Most bleeding and thrombotic complications occurred late during treatment.
